# Self-Help in Institutional Economy

**Published:** 1913-01-18

**Authors:** 


### SELF-HELP IN INSTITUTIONAL ECONOMY.

### IV.—Hospital Gardening and the Supply of Vegetables.

The old hospitals nearly all possessed gardens and supplied themselves with vegetables by utilising to the full their garden acres. Thus, it is recorded that the old general hospital at Vienna, in the reign of its founder, had an extensive garden annexe, from which it obtained both fruit and fresh vegetables. The large hospital at Milan also possessed such a garden, and some of the smaller Italian hospitals yet provide themselves with fresh vegetables out of their own gardens.

In England some hospitals still possess a fair amount of garden space, although none of them, so far as we know, utilise this space—which in the case of some hospitals, as, for instance, the main hospital at Exeter, is of considerable area—to secure a supply of fresh vegetables. The decay of the hospital garden has been due largely to the increase in size of the institutions, which necessitated a corresponding increase in the quantity of vegetables required each day. To obtain such a quantity, again, it was thought that a comparatively large extent of ground was necessary, and only such institutions as possessed private farms could embark on a scheme of self-provisioning. One such scheme was actually tried. In his evidence before the Lords Committee on Hospitals, Mr. Lushington, the then treasurer of Guy's Hospital, stated that an attempt had been made to farm one of the country estates which under Guy's will became the property of the hospital, but that the attempt had been abandoned.

The reasons why it was given up do not seem to be very clear, and one can only surmise that the initial expense and the lack of skilled assistance proved too much for the courage of the governors. The quantity of vegetables needed for a large hospital of the size of the London Hospital or the Edinburgh Infirmary is very great, and it is justifiable to doubt whether amateur gardening on a small scale will prove equal to the task of supplying the daily need. By making use of "intensive" methods of cultivation, the produce of an acre of ground of garden can be more than quadrupled. Such methods, while demanding care and expert knowledge, are very cheap. The initial outlay on "cloches," or bell jars, which may be bought wholesale at from £5 a gross, is not very large, and it has been estimated that with a capital of from £600 to £800 a small farm under good management can be made to be self-supporting. Such a farm will supply a 300-bedded hospital with fresh vegetables practically all the year round, and some profit may be made in addition by the growing of fruit and flowers, which may either be used by the institution or sold in the market. At present hospitals are supplied with vegetables by two chief methods. The one is the contract method, in which the institution contracts with tenderers for a daily supply, the quality of which is ensured by fixing forfeits in case of the supply proving of inferior quality. This method is open to many objections, although it has the great advantage that it frees the institution from all worry so far as the regular supply of vegetables is concerned. Where it is adopted care must be taken to check the daily delivery and to go carefully over the stock supplied. The other and preferable method is for the hospital to appoint its own buyers, who buy the vegetables in the open market every day and who are able to secure the best material at current market prices. These prices necessarily fluctuate, whereas under contract an average price is charged, which is generally a fairly high average and makes allowance for commission charges.

Certain German hospitals, notably those of the Rhine provinces, have recently adopted a modification of the second method by means of which the middleman's commission is avoided and a material economy practised. They buy direct from the grower or importer, not in the market but on the farm, sometimes tendering for a whole crop. The garden produce so purchased is brought direct to the institution, thereby again avoiding intermediate transport charges. In several institutions a special department has been created for the preserving of vegetables by drying or other means, such as pickling—a method very popular in German hospitals, where sauerkraut frequently figures on the ward menu. The drying apparatus used is generally one consisting of sixty shelves, capable of dealing with a fairly large quantity of vegetables. This is heated by a regulating apparatus to 80°, a temperature sufficient to dry most vegetables in a period varying from two to six hours. The drying machine is very simple in construction and easily manipulated. Vegetables preserved in this way retain all their nutritive qualities, and usually also their colour and taste. By centrifugalising the vegetables in the wet state previous to drying them the desiccating process is much quickened, but vegetables subjected to this preliminary treatment are apt to lose something of their flavour, and this method is no longer popular. Desiccated vegetables are greatly reduced in mass—since most green vegetables consist of 90 per cent. of evaporable water—and therefore very convenient for storage; they are largely used in Continental hospitals, but are comparatively little known in English institutions.

Hospital gardening has been practised by many sanatoria and mental hospitals both in this country and abroad. In a series of articles which we published in 1911 we dealt at length with sanatorium gardening, and showed how profitable it could be made under proper management. The experience derived from institutions where such methods of self-help have been in use for a considerable period entirely support our observations. A daily supply of excellent garden produce is secured at prices which compare, on the whole, very favourably with those obtaining in the market; while the institution secures at the same time an agreeable garden. Of course, an essential is that garden space should be available. For that reason a garden attached to the hospital itself is impracticable in most large city hospitals which do not possess the ground. Such institutions, if they attempt gardening as a policy of self-help, must secure an area of ground in the suburbs or in the country, in which case the question of transport and administration is an important one to consider. This is probably why so few hospitals have even attempted to deal with the problem of the vegetable supply in the same radical manner that they have dealt with the milk and meat supply. The initial outlay is heavy, the cost of upkeep is considerable, and the obvious advantages of possessing private vegetable farms are not so great as the advantages of having private milk dairies and private butchers' departments. Again, the method of buying direct from the farmer and eliminating the middleman has effected such a reduction of expenditure that most hospital authorities are content, having adopted this method, to rest on their administrative oars.

Nevertheless, the advisability of starting co-operative farms has been seriously discussed, and it is within the bounds of probability that an endeavour will be made to try the experiment in one or two cases. The advantages of co-operating in this method are very great, and are sufficiently obvious to need no demonstration. The only point which so far opposes the scheme is the fact that most hospitals find the buying in the market or buying from the farm so satisfactory that they do not greatly desire a change, especially if it is to be in the nature of an experiment which will be strenuously opposed by those interested in supplying the institutions with vegetables under existing conditions. When the ultimate ideal of the hospital city is realised, it is clear that hospital farming on a large scale and on a private or co-partnership basis is almost inevitable. Meanwhile, the experience derived from sanatorium and mental hospital gardening will be of the utmost benefit to the institution which is courageous enough to embark on a scheme of independent gardening.

